# Providing NHS staff with height-adjustable workstations and behaviour change strategies to reduce workplace sitting time: protocol for the *Stand More AT* (*SMArT*) *Work* cluster randomised controlled trial

**DOI:** 10.1186/s12889-015-2532-5

**Published:** 2015-12-09

**Authors:** S. E. O’Connell, B. R. Jackson, C. L. Edwardson, T. Yates, S. J. H. Biddle, M. J. Davies, D. Dunstan, D. Esliger, L. Gray, P. Miller, F. Munir

**Affiliations:** Leicester Diabetes Centre, University Hospitals of Leicester, Leicester, UK; School of Sport, Exercise and Health Sciences, Loughborough University, Leicestershire, UK; Diabetes Research Centre, University of Leicester, Leicester, UK; NIHR Leicester-Loughborough Diet, Lifestyle, and Physical Activity Biomedical Research Unit, Leicester, UK; Institute of Sport, Exercise and Active Living, Victoria University, Melbourne, Australia; School of Public Health, The University of Queensland, Brisbane, Queensland Australia; Baker IDI Heart and Diabetes Institute, Melbourne, VIC Australia; Department of Medicine, Monash University, Melbourne, VIC Australia; Department of Epidemiology and Preventive Medicine, Monash University, Melbourne, VIC Australia; School of Exercise and Nutrition Sciences, Deakin University, Burwood, VIC Australia; School of Sport Science, Exercise and Health, The University of Western Australia, Perth, WA Australia; Mary MacKillop Institute for Health Research, The Australian Catholic University, Melbourne, VIC Australia

**Keywords:** Sedentary behaviour, Sit-stand, Workplace sitting, RCT, Physical activity, Behaviour change, Intervention

## Abstract

**Background:**

High levels of sedentary behaviour (i.e., sitting) are a risk factor for poor health. With high levels of sitting widespread in desk-based office workers, office workplaces are an appropriate setting for interventions aimed at reducing sedentary behaviour. This paper describes the development processes and proposed intervention procedures of *Stand More AT* (*SMArT*) *Work*, a multi-component randomised control (RCT) trial which aims to reduce occupational sitting time in desk-based office workers within the National Health Service (NHS).

**Methods/Design:**

*SMArT Work* consists of 2 phases: 1) *intervention development*: The development of the SMArT Work intervention takes a community-based participatory research approach using the Behaviour Change Wheel. Focus groups will collect detailed information to gain a better understanding of the most appropriate strategies, to sit alongside the provision of height-adjustable workstations, at the environmental, organisational and individual level that support less occupational sitting. 2) *intervention delivery and evaluation*: The 12 month cluster RCT aims to reduce workplace sitting in the University Hospitals of Leicester NHS Trust. Desk-based office workers (*n* = 238) will be randomised to control or intervention clusters, with the intervention group receiving height-adjustable workstations and supporting techniques based on the feedback received from the development phase. Data will be collected at four time points; baseline, 3, 6 and 12 months. The primary outcome is a reduction in sitting time, measured by the activPAL^TM^ micro at 12 months. Secondary outcomes include objectively measured physical activity and a variety of work-related health and psycho-social measures. A process evaluation will also take place.

**Discussion:**

This study will be the first long-term, evidence-based, multi-component cluster RCT aimed at reducing occupational sitting within the NHS. This study will help form a better understanding and knowledge base of facilitators and barriers to creating a healthier work environment and contribute to health and wellbeing policy.

**Trial registration:**

ISRCTN10967042. Registered 2 February 2015.

## Background and rationale

Sedentary behaviour has been defined as ‘any waking behaviour characterised by an energy expenditure ≤ 1.5 metabolic equivalents while in a sitting or reclining posture’ [1]. In the past decade or so it has emerged as a risk factor for poor health, often independent of moderate-to-vigorous physical activity (MVPA) [2]. Sedentary behaviour should not be viewed simply as the lack of sufficient exercise, it has been acknowledged as a unique behaviour with its own determinants [1]. High levels of sedentary behaviour and prolonged bouts of sitting have been associated with an increased risk of overweight and obesity, cancer, type 2 diabetes, CVD, and premature mortality [3–10]. These consistent epidemiological findings are supported by research demonstrating positive effects on metabolic regulation of breaking prolonged sitting with bouts of standing and light movement [11, 12]. Moreover, small workplace intervention studies have found that in addition to the metabolic benefits, reduced sitting and increased standing in the workplace is associated with reduced musculoskeletal complaints, improved perceptions of health [13] and reduced fatigue [14]. There is also some evidence to suggest that breaking up prolonged bouts of sitting at work can also improve employee’s productivity [15, 16].

Sedentary behaviours are increasingly prevalent with many adults spending a significant proportion of their waking hours sitting whilst commuting, at their computers working, surfing the internet and watching television [17]. Sedentary behaviour can be reduced by individuals moving from a seated posture to standing or light ambulation resulting in ‘light physical activity’ (LPA) [11]. Reducing sitting time by providing an environment that makes sitting less likely and standing/moving easier could have significant health benefits while affecting large numbers of people and with minimal effort or planning on the part of the individual. A good place to start this cultural shift is in the workplace as many jobs are desk-bound [18] and it has been suggested that around 70–85 % of working hours amongst office, call centre and customer service employees are typically spent sedentary [18, 19].

There has been promising work in small international studies [20–22] demonstrating that the introduction of sit-stand desks can reduce both occupational and leisure sitting time. However, as the majority of these international studies were small and with a short-follow up, larger and longer term studies using a randomised controlled design are needed. One such example is Stand UP Victoria [23], a large-scale, multi-component intervention which aims to reduce prolonged sitting in office-based workers in Australia. However, there is a distinct lack of research on occupational sedentary interventions in the UK [24] and this gap in the evidence needs to be addressed.

The National Health Service (NHS) in the UK is the world’s fifth largest employer with around 1.7 million people employed in 2012. Front line medical staff are a small minority of the overall workforce, with administrative desk-bound jobs making up a large proportion of employees [25]. Furthermore, absenteeism through poor health is a recognised problem within the NHS, while sickness absence rates are highest in support staff on lower pay banding, many of whom are predominately in sedentary desk-bound jobs [26]. The NHS would therefore be the ideal demographic for behaviour change intervention.

This study seeks to build on previous research by robustly evaluating through a cluster randomised controlled trial the effectiveness of a behaviour change intervention focused on reducing occupational sitting in NHS office workers. The behaviour change intervention will provide height-adjustable workstations supplemented by additional behaviour change strategies. This is a two phase study which consists of a development phase to develop the behaviour change strategies, using a community-based participatory research approach, that sit alongside the height-adjustable workstation, and the intervention delivery and evaluation phase to test effectiveness and cost-effectiveness of the intervention.

## Aim

To develop and evaluate an intervention aimed at reducing workplace sitting time within an NHS work force.

## Primary objectives

- To develop an intervention, that incorporates height adjustable workstations, self-monitoring tools and other behavioural chance techniques, aimed at reducing sitting within an NHS workforce
- To evaluate the effectiveness of the developed intervention at promoting reduced occupational sitting time over 12 months

## Secondary objectives

- To investigate whether the developed intervention results in reduced sitting, increased standing time and daily movement over the short- to medium-term (3 and 6 months).
- To investigate whether the developed intervention changes absenteeism and presenteeism, job performance, job satisfaction, work engagement, occupational fatigue, musculoskeletal health, mood/affective states, cognitive ability, quality of life, self-reported workplace sitting, self-efficacy, habit formation and sleep quality over the short, medium and longer term.
- To determine the cost-effectiveness of the intervention from the employer’s perspective.

## Methods

This study will be conducted over two phases: intervention *development* and intervention *delivery and evaluation*. Ethical approval has been sought and obtained from Loughborough University and Loughborough University will act as study sponsor.

### Phase 1: Intervention development

During the development phase of the SMArT Work project, detailed information will be collected, from focus groups, to gain a better understanding of the most appropriate and acceptable strategies at the environmental, organisational and individual level that can sit alongside the provision of height-adjustable workstations to support an occupational focus on sitting less and standing more among office-based employees.

#### Theoretical basis

Behaviour change interventions grounded in theory tend to be more effective [27].

**Fig. 1:**
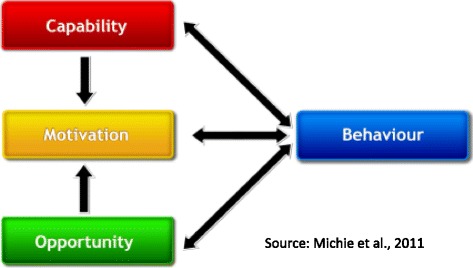
The COM-B Model

The development of this intervention will take a community-based participatory research (CBPR) approach using the Behaviour Change Wheel (BCW) [28, 29]. At its core, the BCW has a model of behaviour change known as the COM-B model, with the central tenet that behaviour is an interacting system comprising of the three core components of *capability*, *opportunity*, and *motivation* (see Fig. 1). Any change in behaviour would involve the manipulation of one or more of these components to put the system into a new configuration. Each component is further broken down into two distinct categories: *psychological* and *physical* capability, *social* and *physical* opportunity, and *automatic* and *reflective* motivation. Focus groups or interviews must be conducted where discussions about the barriers and facilitators of the target behaviour identify which COM-B components the intervention should focus on in order to elicit the desired change. We will be using the Theoretical Domains Framework (TDF) – a variant of the COM-B model which subdivides the COM-B components into linked behavioural domains – to prompt discussions about sedentary behaviour in the workplace.

#### Participant recruitment

Participant recruitment will be coordinated via the research team at the Leicester Diabetes Centre. We currently hold a database of office units within the University Hospitals of Leicester NHS Trust and will promote this study to them initially through the use of the Trust’s intranet and emails to department managers. This will be followed up with a face-to-face presentation/meeting if necessary. A stratified sample of NHS staff (e.g. employees, managers, gender, job role) will be targeted where possible. NHS staff will be sent an invitation letter, participant information sheet (PIS) and reply slip. Those who are interested in being involved will be followed up with a phone call or email to arrange either a face-to-face interview or focus group.

#### Focus groups

TDF-based focus groups:

- NHS office-based staff will be invited to take part in TDF-based focus groups. The focus group schedule will follow the theoretical constructs of the TDF in order to inform the development of the intervention. Each theoretical construct, or ‘domain’, relates to a COM-B component – all of which need to be present for any behaviour to occur. The focus groups will identify the barriers and facilitators to reducing sitting at work and ascertain which COM-B component(s) should be the primary focus of the intervention strategies.
- As well as the capability, opportunity, and motivation to reduce sitting work, it is anticipated that focus groups will discuss the following: 1) staff/organisation readiness to change, 2) perceptions of sedentary behaviour in the workplace, 3) possible options for behaviour change strategies to reduce/break up sitting through the working day - this will cover environmental strategies such as central waste bins and printers, organisational strategies such as displaying posters around the offices about the negative impacts of prolonged sitting, and personal strategies such as setting reminders to get up every 30 min, and 4) preferences for height-adjustable work stations.
- Demographic data will be collected at the focus groups including age, gender, job type, and working hours via a short questionnaire. We anticipate conducting up to eight focus groups in a representative sample (of between 4–8 participants in each focus group). However, if we reach the point of data saturation where no new themes are emerging then data collection will cease.

Self-monitoring device trial and feedback focus groups:

- Following the TDF-based focus groups, a sub-sample of participants who agree will be given the opportunity to trial some of the electronic self-monitoring devices that are currently available for providing feedback on sedentary behaviour and physical activity (e.g., Lumoback, Darma cushion, activPAL VT). Each participant will be given two or three devices to trial. Whilst trialling each device participants will be asked to complete a short questionnaire about the ease of use, usefulness for self-monitoring and whether it encouraged a reduction in sitting time. Once participants have trialled their devices, further focus groups will be conducted using a flexible topic guide (and questionnaire feedback as prompts for discussion) to gain more detailed feedback on the devices. Although the focus of each discussion may be slightly different depending on the device trialled, topics will generally focus on how much the device helped them towards achieving their goal, how useful it was to monitor sitting and movement time, ease of use of the device in relation to charging, syncing data and accessing feedback on sedentary behaviour, comfort, usefulness of feedback and whether it was user friendly, provision of tailored feedback and ability to goal set, any difficulties with using the tool, and overall willingness to use the tool for longer periods of time. This information will enable us to decide on the most appropriate self-monitoring device to provide during Phase 2.

#### Sample size

As this is a qualitative study, a formal sample size calculation has not been conducted. Data collection will cease when data saturation (i.e. when no new themes are emerging) has been reached, this is expected to be no more than 60 participants [30].

#### Analysis and intervention development

Template analysis [31] will be conducted using a priori themes to identify which of the 14 TDF domains play an important role and might facilitate the target behaviour. These will then be used to ascertain the relevance of each of the COM-B components to a sedentary behaviour intervention, which, in turn, map onto a selection of intervention functions (IF) and behaviour change techniques (BCT) within the 93-item behaviour change taxonomy [32]. For example, domains of *knowledge* (capability) and *environmental context and resources* (opportunity) might be found to facilitate the target behaviour of sitting less at work. Appropriate intervention functions for increasing knowledge and adapting the environment would be *education* and *environmental restructu*ring, resulting in an intervention consisting of BCT’s appropriate for these IF’s and identified TDF domains, such as providing *feedback* and *information* (knowledge) while using *prompts*/*cues* and *adding objects to the environment* (environmental context and resources).

### Phase 2: Intervention delivery and evaluation

Subsequent to the development phase, the developed intervention will be delivered and evaluated over a 12-month period.

#### Design

A two-arm, cluster RCT will be undertaken to evaluate the intervention. Participants will be randomised by cluster to receive the intervention or act as the control group and maintain their usual working environment. The Consolidation Standards of Reporting Trials (CONSORT) statement for cluster RCTs will be used to conduct, analyse and report this study.

#### Study setting

This trial is targeting office-based NHS workers. Participants will be recruited from within the University Hospitals of Leicester (UHL) NHS Trust main sites (Leicester Royal Infirmary, Glenfield Hospital and Leicester General Hospital, all in Leicestershire, UK).

#### Sample size

To detect a difference in objectively measured sitting time of 60 min, assuming a SD of 60 min, 90 % power and 5 % alpha we would require 22 participants per arm, if the trial was individually randomised. As this is a cluster randomised trial, this sample size needs to be inflated to take into account the clustering. Assuming an average cluster size of 13 (varying between 3–50, therefore CV = 0.9) and an intraclass correlation coefficient of 0.05 gives a design effect of 2.13, resulting in 65 participants from 5 clusters per arm. To conform to best practice we have increased the sample size to take into account both drop out at the cluster and participant level. Therefore we will recruit 17 participants per cluster, this allows for the primary outcome data to be unavailable for 25 % of participants. Increasing the number of clusters by one per arm therefore gives a total of 7 clusters per arm each recruiting 17 participants on average (7*17*2 = 238), giving 238 participants in total. Exact cluster sizes may be modified after the first phase of the project (focus groups, intervention development).

#### Randomisation

Randomisation will occur after all baseline assessments and data is collected. Clusters will be randomised 1:1 to either intervention or control groups stratified by office size with a block size of 4. Clusters will be randomised by an independent statistician.

#### Inclusion criteria

Participants who meet the following criteria will be eligible:

- are office-based (≥75 % of seated working hours, excluding mandatory work breaks)
- work within UHL NHS Trust,
- aged between 18–70 years of age,
- have the capability of standing,
- work at least three days a week at the same desk.

#### Exclusion criteria

NHS staff that are not predominantly office-based (<75 % seated working hours), work less than three days at one desk (i.e. cross-site workers), or will not be working for the full duration of the study (i.e. retirement, term-time only, maternity leave) will be excluded from taking part. If participants are unable to read and understand English or provide full informed consent they will be ineligible for the study. If participants are severely incapacitated with existing musculoskeletal conditions which restrict them from standing, they will be excluded from the study.

#### Recruitment procedures

**Fig. 2:**
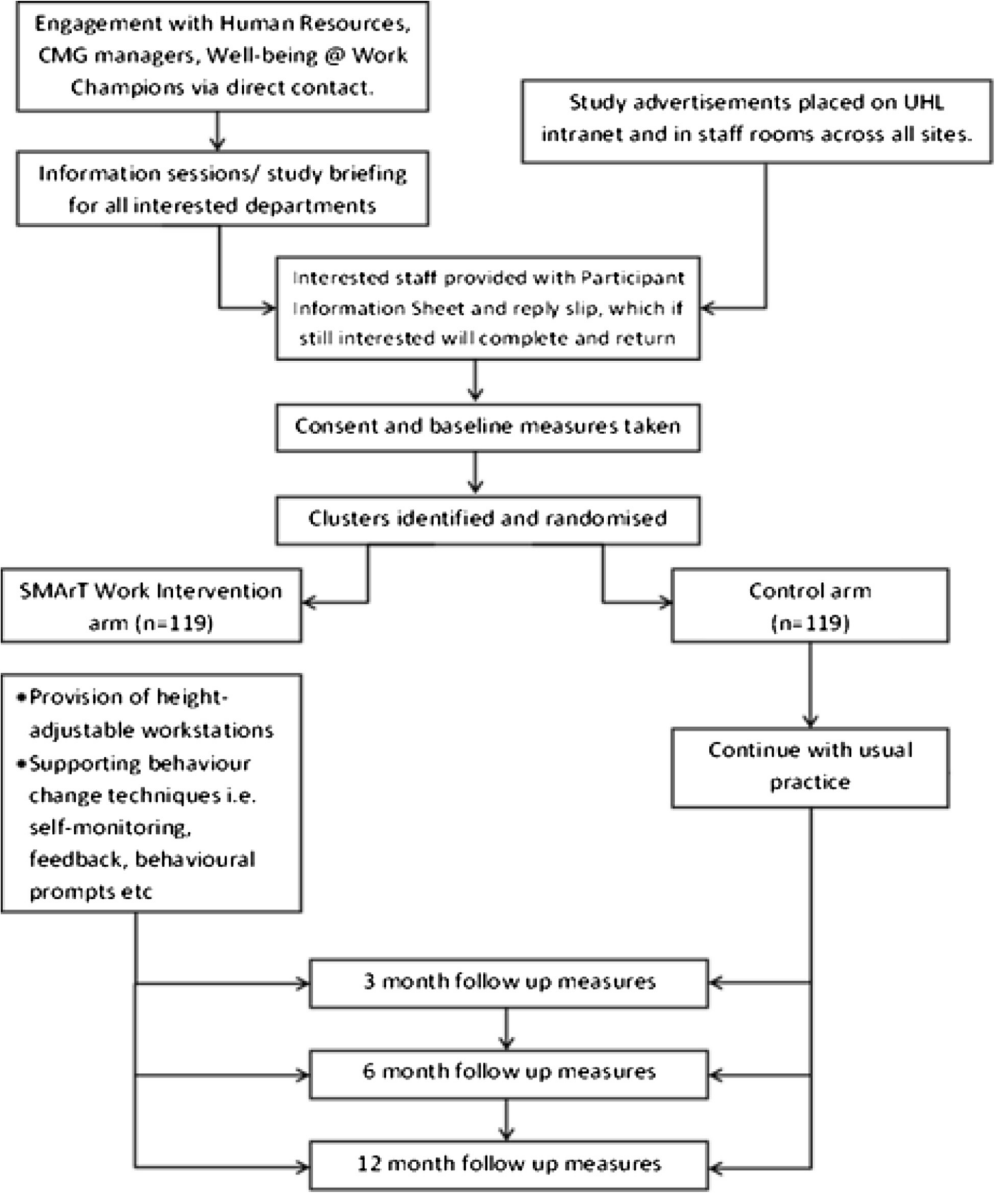
Recruitment Flow

Figure 2 demonstrates the flow of the study participants from start to finish. All procedures are explained in the following sections.

#### Study clusters

In order to control contamination, or minimise interaction between the treatment and control group, randomisation will be done on a cluster level. The nature of the study clusters will depend on recruitment numbers, but may be distinct office space, floors or whole departments. In order to identify study clusters (i.e. a small distinct group randomised to either control or intervention condition with multiple clusters making up each arm of the trial) we aim to work closely with the Well-being @ Work Champions within the University Hospitals of Leicester NHS Trust to advertise the study to departmental managers across all sites. Adverts will also be placed in staff rooms and on the staff intranet to promote the study. The research team will conduct information sessions for departments on all sites outlining the study requirements and expectations from both individual employees and the team. In addition, appropriate management support will be sought for any environmental components of the intervention to be incorporated into the office workspace and for the behavior-change elements to be conducted during work time. Managers will be asked to express unreserved commitment to having their employees participate in the study. Consenting clusters will be randomised to either the intervention or control arms of the trial.

#### Individual participants

Employees within identified clusters will be recruited via various internal advertisements including study posters displayed in staff rooms, email messages from managers and notifications on the staff intranet and social media sites. All of these will advertise the contact details of the study team whereby interested participants can request more information about the study. An invitation letter and a participant information sheet will be delivered to them via post or email. Participants will be asked to complete a reply slip if they wish to be involved in the study. Researchers will check each participants eligibility from the details entered on the reply slip and will contact eligible participants to organise a convenient date to consent them into the study and take their baseline measurements. To ensure individual participants are included in the study and are part of a study cluster, researchers will approach their office to make an informal information presentation about the study to spark interest in fellow co-workers of the individual participant.

#### Intervention procedures

The Behaviour Change Wheel [28] will be used to guide our intervention. Therefore, we will include behaviour change strategies at three levels: environmental, organisational and individual. The focus groups in the development phase of the SMArT Work project will allow the team to design more specific organisational and individual intervention strategies.

#### Environmental

A core element of the intervention is the provision of a height-adjustable workstation to those allocated to the intervention arm of the study. Participants will get the opportunity to choose a height-adjustable workstation (within a set budget), which they believe will be the most suited to them and their working environment. Short in person training sessions on the use of the desks and elements of health and safety as well as educational flyers promoting the benefits of a height adjustable workstation will also be provided for staff.

#### Organisational

In order to provide further opportunities and motivation to break up prolonged sitting during the working day, additional strategies to the height-adjustable workstation may be used. Evidence-based strategies which may be implemented include the display of signs promoting the use of their desk, small-scale environmental restructuring (e.g., removal or relocation of individual printers and waste bins), inclusion of standing in meetings, provision for lunch time walking or internal competitions. These are just examples and the exact organisational strategies will be decided upon following the TDF-based focus groups in Phase 1.

#### Individual

Additional evidence-based behaviour change strategies will also be used to enhance the likelihood of adoption and maintenance of reduced workplace sitting. Strategies that have been identified as particularly effective in physical activity behaviour-change include self-monitoring, feedback and behavioural prompts [33–35]. In this instance, we will provide participants with an electronic self-monitoring device that will allow them to monitor their sitting behaviour. The exact device used will be decided upon following the self-monitoring trial and focus groups in phase 1. Further examples of individual intervention strategies that could be implemented if identified during the development phase could be goal setting, action planning, and social comparisons.

### Data collection

Data will be collected at four time points (unless stated otherwise); baseline, 3, 6 and 12 months. Trained researchers, following standard operating procedures, from University of Hospitals of Leicester, University of Leicester and Loughborough University will collect data.

#### Objectively measured sitting and physical activity

Participants will wear an activPAL^™^ micro monitor on their thigh 24 h/day for 7 days. Both work and leisure-time sitting will be assessed to see if any reduction at work is compensated for by more sitting at home. Participants who provide an adequate number of valid days will receive a £5 voucher at each data collection time point. The activPAL has been found to be a valid and reliable measure of sitting, standing, stepping and postural transitions in adults [36–40]. The following variables will be calculated from the device:

1. Mean minutes spent sitting,
2. Mean minutes spent standing,
3. Mean minutes spent stepping
4. Mean number of sit-to-upright transitions
5. Mean minutes spent in prolonged (e.g., bouts of ≥30 min) sitting

These variables will be calculated across the total waking day and separately by time at work and leisure time.

Alongside the activPAL monitor, participants will be asked to wear a wrist worn accelerometer 24 h/day for 7 days to capture time spent in different intensities of physical activity. In addition, participants’ office/desk dwell time will be measured using proximity-based location devices. To the authors’ knowledge, this will be the first use of this innovative technology in a standing desk study. Deploying proximity-based location devices will allow for the precise and burden-free quantification of the amount of time participants spend at their desk (both in the control and intervention group). Whilst wearing the devices, participants will be asked to complete a short diary each day to note the time they went to bed, went to sleep, woke up and got out of bed each day, hours they worked each day, as well as recording any periods throughout the day if they removed the devices (i.e., activPAL and wrist accelerometer).

#### Demographic and anthropometric measures

During their baseline visit, participants will be asked about their age, ethnicity, smoking status, dependents, marital status, education level, current job role and grade, working hours, length of time in post in the NHS, postcode and household composition. At each follow up visit, participants will be asked if there has been any change in these measures. At baseline and each follow up visit, participants will have their height, weight, waist circumference (WC), blood pressure (BP) and body fat percentage measured. Height will be measured to the nearest centimetre using a Leicester portable height measure. Weight, in kilograms, and body composition will be measured using a Tanita Body Composition Analyser (Tanita UK Ltd, Middlesex, UK). Participants will be asked to remove shoes, socks and heavy outerwear clothing and to ensure their pockets are empty before stepping on to the scales. Body mass index (BMI) will be calculated by the scales as kg/m^2^. WC will be measured to the nearest centimetre (cm) using a standard anthropometric tape measure, with the tape measure being placed around the abdomen midway between the uppermost border of the iliac crest and the lower edge of the chest (thorax) formed by the bottom edge of the rib cage. BP will be assessed using an Omron M6 automated blood pressure monitor (Omron Healthcare Europe, Hoofddorp, Netherlands). Participants will be asked to sit quietly and relax prior to having their BP measurements taken and three readings will be taken.

At baseline and 12 months follow up participants will have point of care testing (POCT) for the measurement of HbA1c, triglycerides, glucose, total cholesterol, HDL cholesterol and calculated LDLc. Capillary blood samples will be taken from each participant using the finger prick method. The CardioChek® system, which is a portable hand-held device that requires between 15–40 μL (millions per microliter) of blood taken using a finger-stick, will be used for these measurements. No blood will be stored and all blood contaminated testing sticks will be deposed of appropriately. All participants will receive feedback on these results. If any of the values for these measures appear to be outside of the normal range or raise concern with the research team, results will be checked by a medic. Participants will be advised to contact their GP and a letter will be sent to their GP detailing the results.

#### Work-related health measures

Various measures will be employed in order to characterise work-related health:

- Job performance [41] and job satisfaction [42] will be measured using single-item 7-point likert scales, and participants will also be asked to indicate the extent to which they intentionally changed their work priorities and objectives to accommodate the change from sitting behaviour (6-point fully anchored scale).
- Work engagement (characterized by vigour, dedication, and absorption) will be measured using the Utretcht Work Engagement Scale (UWES) [43]; a multi-item 7-point likert scale.
- Occupational fatigue will be measured using the Need for Recovery (NFR) Scale [44].
- In order to assess self-reported ratings of symptoms most often encountered in an occupational setting the Standardised Nordic Questionnaire (SNQ) for the analysis of musculoskeletal symptoms [45] will be used.
- Work-related cognitive function will be assessed using an experimental cognitive test battery including measures for memory recall [46], verbal fluency [47], attention [48], information processing (digit-symbol substitution task) [49, 50] and executive function [51].
- Data on sickness absence will be collected using both self-report and from employer records and include frequency and duration of self-certified and certified sickness. Reasons for sickness absence will also be recorded. Data on sickness absence will be collected for 12 months prior to the intervention and for the 12 months of the intervention and follow-up period.
- Presenteeism will be assessed both by using the 8-item Work Limitations Questionnaire [52] that asks participants to rate on a six-point Likert scale how their health has affected aspects of their work in the past two weeks, and the Work Productivity and Activity Impairment Questionnaire (WPAI-GH 2.0) [53].
- Participant's sleep duration and quality will be assessed using the Pittsburgh Sleep Quality Index (PSQI) [54].
- Finally, to assess participant’s perceptions of work and job demands, the Health and Safety Executive Management Standards Indicator Tool (HSE MSIT) [55], which uses a 5-point likert scale for participants to reflect their work over the last 6 months, will be administered.

#### Psycho-social measures

A mix of qualitative and quantitative psychosocial measures will be employed at baseline, 3, 6, and 12 months to identify behavioural self-efficacy (based on the application of the Theory of Planned Behaviour [56], acceptability and awareness of environmental and organisational change, strength of ‘habit’ in workplace behaviours, including sitting (modified Self-Report Habit Index) [57]; while mood/affective states will be measured using the Multiple Affect Adjective Checklist-Revised [58]. A general assessment of quality of life will be conducted using the WHOQOL-BREF [59].

#### Subjective sitting time

Participants will be asked to complete the Workplace Sitting Breaks Questionnaire (SITBRQ) [60], a 2-part question, asking about the amount of breaks from sitting an individual will take during a typical hour at work and roughly the amount of time spent in short physical activity breaks across a typical work day.

For a complete list of outcomes and scheduled measurements, see Table 1.

**Table 1:** Schedule of the outcome measures

Measure	Baseline	3 months	6 months	12 months
Objective siting time and physical activity (activPAL and wrist accelerometer)	✓	✓	✓	✓
Office/desk dwell time (proximity-based location devices)	✓	✓	✓	✓
Job performance	✓	✓	✓	✓
Job satisfaction	✓	✓	✓	✓
Work engagement (UWES)	✓	✓	✓	✓
Occupational fatigue (NFR)	✓	✓	✓	✓
Habit (SRBAI)	✓	✓	✓	✓
Musculoskeletal symptoms (SNQ)	✓	✓	✓	✓
Cognitive function tests	✓	✓	✓	✓
Self-reported workplace sitting (SITBRQ)	✓	✓	✓	✓
Presenteeism (WLQ and WPAI-GH 2.0)	✓	✓	✓	✓
Self-efficacy	✓	✓	✓	✓
Mood/affective states (MAACL-R)	✓	✓	✓	✓
Quality of Life (WHOQOL-BREF)	✓	✓	✓	✓
Sleep Quality (PSQI)	✓	✓	✓	✓
Work and Job Demands (HSE MSIT)	✓		✓	✓
Self-reported sickness absence	✓			✓
Sickness absence via employee records	✓			✓
Anthropometric measures	✓	✓	✓	✓
Blood samples	✔			✔

#### Cost analysis

Cost effectiveness will be undertaken following guidelines from the World Health Organisation (WHO) [61]. Programme input costs will be calculated based on the time cost of additional organisational meetings and adjustment of workspace, the cost of desks and physical changes to the workspace and any disruption to normal working patterns, including reasonable allocation of (joint) costs imposed on other activities as a result of the planned changes. Any costs associated with subsequent additional arrangements required to ensure that the process of changed work patterns proceeds would be included. Such costs can then be compared across the control and treatment groups for any array of measures of the output of the intervention such as sickness absence and job performance. Costs will be suitably discounted across time.

#### Process evaluation

A process evaluation will be undertaken to understand the influence of SMArT Work on the study outcomes, to ascertain what elements of the intervention were favoured by the members of staff and to help inform future development. We will use a variety of techniques to gather information on recruitment, data collection, deviations from the protocol, delivery of the intervention and data on environmental factors which may impact on the study.

Interviews will be conducted with a subset (~20) of intervention participants at 12 months to gain additional insight into the perceptions of those that received the intervention and identify what aspects of the intervention worked for them, what was useful and what was not. A topic guide will be used to facilitate the process of data collection, but this will be used flexibly with scope for discussion of additional relevant topics that may arise. Interviews will be audio recorded and transcribed verbatim. Analysis will be iterative and informed by the constant comparative method. Preliminary open codes will be generated from the first few interviews. Ongoing analysis will lead to the development and refinement of the codes into a coding framework. Analysis will be facilitated using a fully-licensed custom software application (NVivo8, a qualitative data-indexing package).

We will also provide participants with disposable cameras to take photos of their workstation and office space at each time point in order to inform a picture content analysis. Such procedures will be carried out for all implemented intervention strategies, as well as work-related meaningful events (contractual changes, managerial/staff changes, policy changes, workload changes).

#### Statistical analysis

A statistical analysis plan will be written and agreed by all investigators before the final data become available for analysis. Cluster and participant level characteristics will be compared by group allocation, using either means (SD) or medians (IQR) for continuous variables, and counts and percentages for nominal variables. The primary outcome, occupational sitting time at 12 months, will be analysed initially on a complete case basis. To adjust for clustering, sitting time will be analysed using generalised estimating equation models with an exchangeable correlation structure and an identity link with a normal distribution. The models will include allocated group and baseline value as covariates. All secondary outcomes, including sitting time at 3 and 6 months, will be analysed using the same method. Sensitivity analyses will be carried out for the primary outcome. The analysis will be repeated imputing any missing values for sitting time using multiple imputation (ITT).

## Discussion

There is accumulating evidence demonstrating the health risks of high levels of sedentary behaviour, with substantial epidemiological evidence illustrating the high proportion of time spent in sitting behaviours in office-based workers. To address excessive sitting as a potentially independent health risk, high quality intervention studies are necessary for determining feasibility, effectiveness and sustainability of these interventions for reducing workplace sitting time. With a lack of large scale, long term RCTs, the primary aim of the SMArT Work trial is to develop a workplace intervention to reduce prolonged sitting over 12 months.

The strengths of the project are the community based participatory research approach in the development of a randomised controlled trial. The robust study design has baseline, 3, 6, and 12 month follow up to assess short, medium and long term behaviour. Additionally, a process evaluation, including a cost analysis, will be an integral part of this trial to ascertain not only if the intervention is effective in reducing occupational sitting time, but also to understand why the intervention is, or is not, feasible and sustainable for future uptake in the NHS or other employers. Finally, this study uses an objective measure of sedentary behaviour for its primary outcome.

The results of this intervention will provide insight for future studies and offer evidence for policy guidelines around workplace health and well-being.

## Abbreviations

BCT: Behaviour change technique
BCW: Behaviour Change Wheel
BMI: Body mass index
BP: Blood pressure
CBPR: Community-based participatory research
COM-B: Capability, opportunity, motivation - behaviour
CONSORT: Consolidation Standards of Reporting Trials
CVD: Cardiovascular disease
GP: General practitioner
HSCIC: Health and Social Care Information Centre
HSE MSIT: Health & safety executive management standards indicator tool
IF: Intervention function
IQR: Interquartile range
ITT: Intention to treat
LPA: Light physical activity
MAACL-R: Multiple affect adjective checklist - revised
MVPA: Moderate to vigorous physical activity
NFR: Need for recovery scale
NHS: National Health Service
POCT: Point of care testing
PSQI: Pittsburgh Sleep Quality Index
RCT: Randomised controlled trial
SBRN: Sedentary Behaviour Research Network
SD: Standard deviations
SITBRQ: Workplace Sitting Breaks Questionnaire
SNQ: Standardised Nordic Questionnaire
SRBAI: Self-report behavioural automaticity index
TDF: Theoretical Domains Framework
UHL: University Hospitals of Leicester
UWES: Utrecht work engagement scale
WC: Waist circumference
WHO: World Health Organisation
WHOQOL BREF: WHO quality of life - brief
WLQ: Work Limitations Questionnaire
WPAI-GH: Work productivity and activity impairment questionnaire: general health
